# Liver-targeting iron oxide nanoparticles and their complexes with plant extracts for biocompatibility

**DOI:** 10.3762/bjnano.15.125

**Published:** 2024-12-11

**Authors:** Shushanik A Kazaryan, Seda A Oganian, Gayane S Vardanyan, Anatolie S Sidorenko, Ashkhen A Hovhannisyan

**Affiliations:** 1 Department of Medical Biochemistry and Biotechnology, Russian-Armenian (Slavonic) University, Hovsep Emin St 123, Yerevan, Armenia; 2 Yerevan State Medical University After M. Heraci, Department of Biochemistry, Koryun St 2, Yerevan, Armenia; 3 Ghitu Institute of Electronic Engineering and Nanotechnologies of Technical University of Moldova, Chisinau, Moldova

**Keywords:** biocompatibility, hepatotoxicity, iron oxide (Fe_3_O_4_) nanoparticle, rutin, *Teucrium polium* (*T. polium*)

## Abstract

Thanks to their simple synthesis, controlled physical properties, and minimal toxicity, iron oxide nanoparticles (Fe_3_O_4_ NPs) are widely used in many biomedical applications (e.g., bioimaging, drug delivery, biosensors, diagnostics, and theranostics). However, the use of NPs does not preclude the possibility of selective toxicity and undesirable effects, including accumulation in tissues and direct interaction with specific biological targets. This study evaluated the biocompatibility of Fe_3_O_4_ NPs, *Teucrium polium* (*T. polium)* extract, rutin, and the corresponding complexes on the liver tissue of healthy white Wistar rats. The impact profile of the synthesized Fe_3_O_4_ NPs (15 ± 4 nm), rutin, *T. polium* extract, and their complexes on biochemical markers of liver function (ALT, AST, ALP, GGT, HDL, LDL, total cholesterol, total protein, and albumin) and morphological indicators of rat liver was investigated. Fe_3_O_4_ NPs, rutin, and *T. polium* extract do not show direct hepatotoxicity when administered intraperitoneally to rats, unlike their complexes. All agents exert a hypolipidemic effect by lowering LDL, despite maintaining the synthetic functions of the liver. Fe_3_O_4_ NPs increase the activity of GPO, which is associated with their peroxidase-like properties. A multifaceted and diverse mechanism of action of all studied samples on the liver of Wistar rats was identified.

## Introduction

Leveraging nanotechnology, personalized medicine, and interdisciplinary collaboration is essential for overcoming the complex challenges associated with human health. Nanotechnology offers great opportunities in medicine because of the physicochemical properties at the nanoscale. There are efforts to apply unique quantum phenomena at the nanoscale in the fields of medicine, biomedical sciences, bioengineering, food technology, biochemistry, biophysics, and other disciplines within biology and medicine [1–5]. The development of nanotechnology has provided resources for various applications in the medical field, leading to significant advances in diagnosis, biological detection, therapy, and drug delivery [6–9]. An interdisciplinary approach is to integrate advances in biotechnology, nanomaterials, biomedical robotics, and genetic engineering into the broader field of nanomedicine. On a larger scale, the application of nanotechnology in medicine enhances efficiency, accelerates processes, and improves functional performance in most biological and chemical reactions involved in the production of medical materials [10–13].

Magnetic nanoparticles (MNPs), such as iron oxides, not only exhibit superparamagnetism and high magnetic susceptibility, they also possess unique physical properties, biocompatibility, stability, and other important qualities [14–15]. Iron oxide NPs, because of their minimal toxicity, are considered the most preferred agents for studying various biomedical applications [16]. There are many studies proving the biocompatibility of iron oxide NPs, and because of their unique properties Fe_3_O_4_ NPs have great potential for commercial use and have already found applications in biomedicine, such as magnetic resonance imaging (as contrast enhancement agents), targeted drug or gene delivery, tissue engineering, biological fluid detoxification, hyperthermia, biological sensing, nanozymes, and cell labeling [17–22]. Their biocompatibility and stability fill the niche of applications that require properties unattainable by organic materials. Size control, prevention of aggregation through coating, specific interactions and dispersion, and the ability to penetrate cellular and tissue barriers all give iron oxide MNPs an advantage over other metallic nanoparticles. Because of their small size, nanoparticles have a high surface-to-volume ratio, making them more appealing. However, since the large surface area provides numerous active sites for interactions, it can also lead to adverse reactions. The toxicity of MNPs depends on various factors such as size, shape, structure, surface modification, concentration, dosage, biodistribution, bioavailability, solubility, immunogenicity, and pharmacokinetics [23–24]. Their use in some clinical applications is limited by low solubility and toxicity effects; as of May 2024, the website clinicaltrials.gov listed data on the development of 51 clinical protocols involving iron oxides NPs [25–27]. Surface chemistry and delivery route of MNPs affect their biodistribution patterns and circulation time in the body [28]. It is known that MNPs larger than 200 nm are captured by the spleen through mechanical filtration, while MNPs smaller than 10 nm can be eliminated via renal clearance. Therefore, the 10–100 nm range is considered optimal for administration in specific applications [29]. The biodistribution patterns of these particles have been identified as 80–90% in the liver, 5–8% in the spleen, and 1–2% in the bone marrow [30].

One of the major organs where nanoparticles are likely to accumulate, depending on the route of administration, is the liver [31–33], where Kupffer cells can quickly uptake large nanoparticles (>100 nm). Smaller nanoparticles (<100 nm) are captured in the space of Disse, from where, if suitably functionalized, they can also accumulate in hepatocytes [34]. It has been shown that Fe_3_O_4_ NPs functionalized with salicylic acid (35 mg/kg body weight) can accumulate in the liver and exhibit hepatotoxicity at a cumulative dose of more than 244 mg/kg over 28 days of exposure [35].

By controlling the physical properties of nanoparticles, it is possible to regulate their delivery and sequestration processes [36–39]. Depending on the size, coating, and duration of exposure, they can exhibit hepatotoxicity and cause inflammatory reactions [31,40–43]. Askri et al. demonstrated the weakness of the antioxidant barrier against these iron nanoparticles [31]. When Fe_3_O_4_ NPs accumulate in lysosomes and release iron ions from their structure, this leads to the dysfunction of mitochondria, lysosomes, the Golgi apparatus, and the endoplasmic reticulum [44–45]. Wu and colleagues found that Fe_3_O_4_ NPs up to 5 nm in size can penetrate cells and initiate the Fenton reaction, resulting in the formation of genotoxic ^•^OH radicals [20]. Moreover, iron overload in cells can lead to ferroptosis [46–47]. Fe_3_O_4_ NPs larger than 5 nm are unable to participate in these mechanisms [20].

Thus, the evaluation of the biocompatibility and hepatotoxicity of Fe_3_O_4_ NPs is relevant for their potential biomedical applications. The combination of phytotherapy with nanotechnology can enhance the pharmacological effectiveness by increasing the selectivity of action and reducing risks through lower dosages. Such approaches appear very promising, but it is essential to consider the risks of nanotoxicology, that is, possible undesirable side effects of nanoparticles.

Combining phytopreparations with biocompatible nanoparticles allows for the delivery of biologically active phytocomponents to the target site with a lower likelihood of their biotransformation, significantly reducing the therapeutic dose of these agents [37,48–49]. The NPs themselves, possessing a certain spectrum of biological activities, contribute to the overall therapeutic and/or prophylactic effect. Therefore, the combined use of biocompatible NPs and medicinal plant extracts makes it potentially possible to achieve breakthroughs regarding new approaches to the treatment and prevention of diseases. The development of such complexes is a relevant issue in biomedicine with significant potential for practical application.

In this work, we studied the biocompatibility and hepatotoxicity of Fe_3_O_4_ NPs both individually and in combination with plant extract from *Teucrium polium (T. polium)* and its active component rutin on the liver of healthy white Wistar rats.

## Results

### Effects on liver function

Previous studies have identified pronounced biocompatible properties in a 70% ethanol extract of *T. polium*, with a total flavonoid content (TFC) value of 26.34 ± 0.11 µg/mL and an IC_50_ value of 1.40 ± 0.05 mg/mL; in addition, rutin (3.74 µg/mL) and genistein (0.21 µg/mL) were detected via HPLC [50]. In this study, we synthesized by a chemical method monocrystalline, round Fe_3_O_4_ NPs with a diameter of 15 ± 4 nm [50–51]. Interaction of NPs with the plant extract and rutin leads to the formation of complexes, as demonstrated by spectral analysis [50].

**Figure 1:**
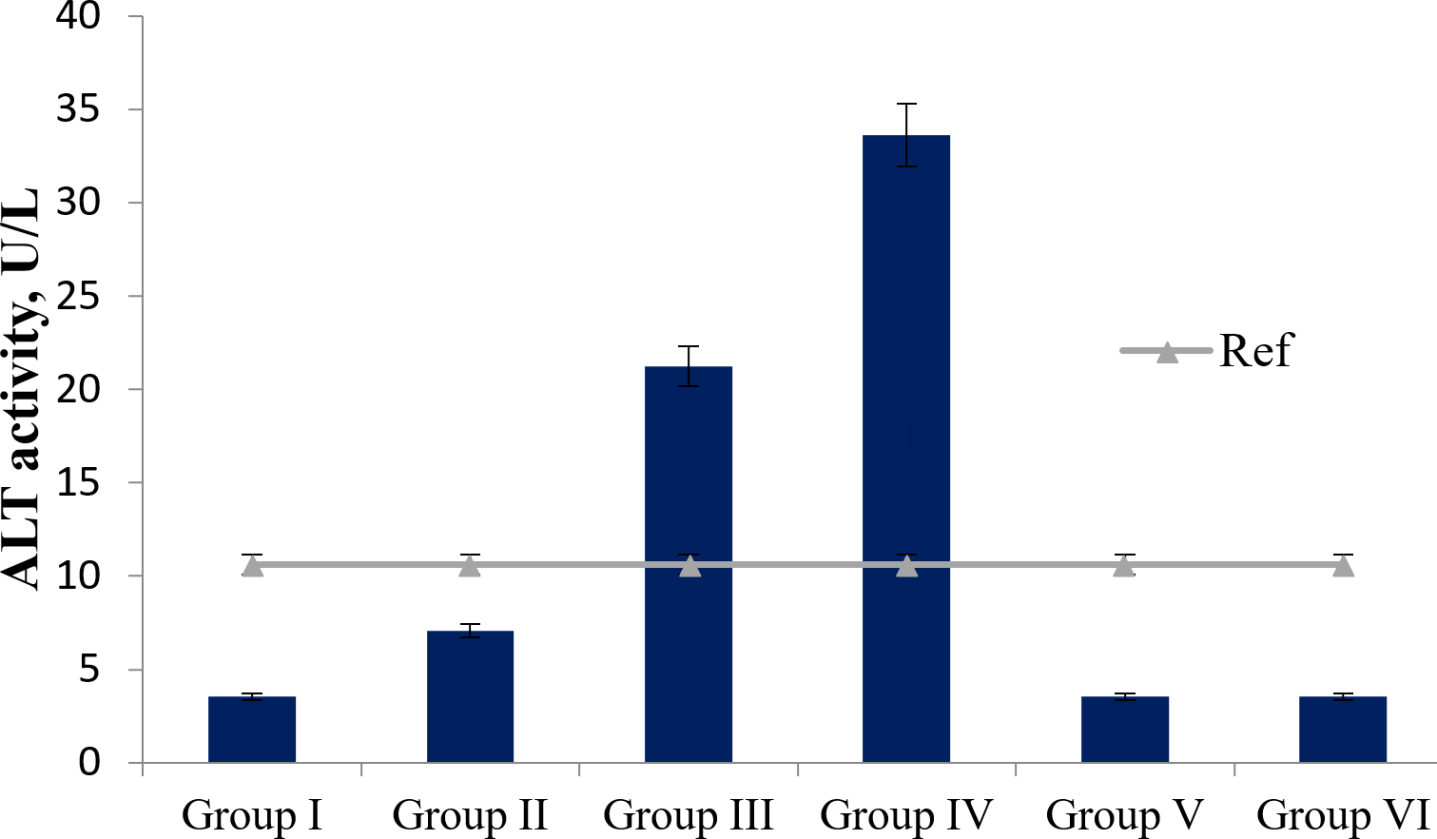
ALT activity (U/L) in the plasma of experimental animals (*p* < 0.05).

**Figure 2:**
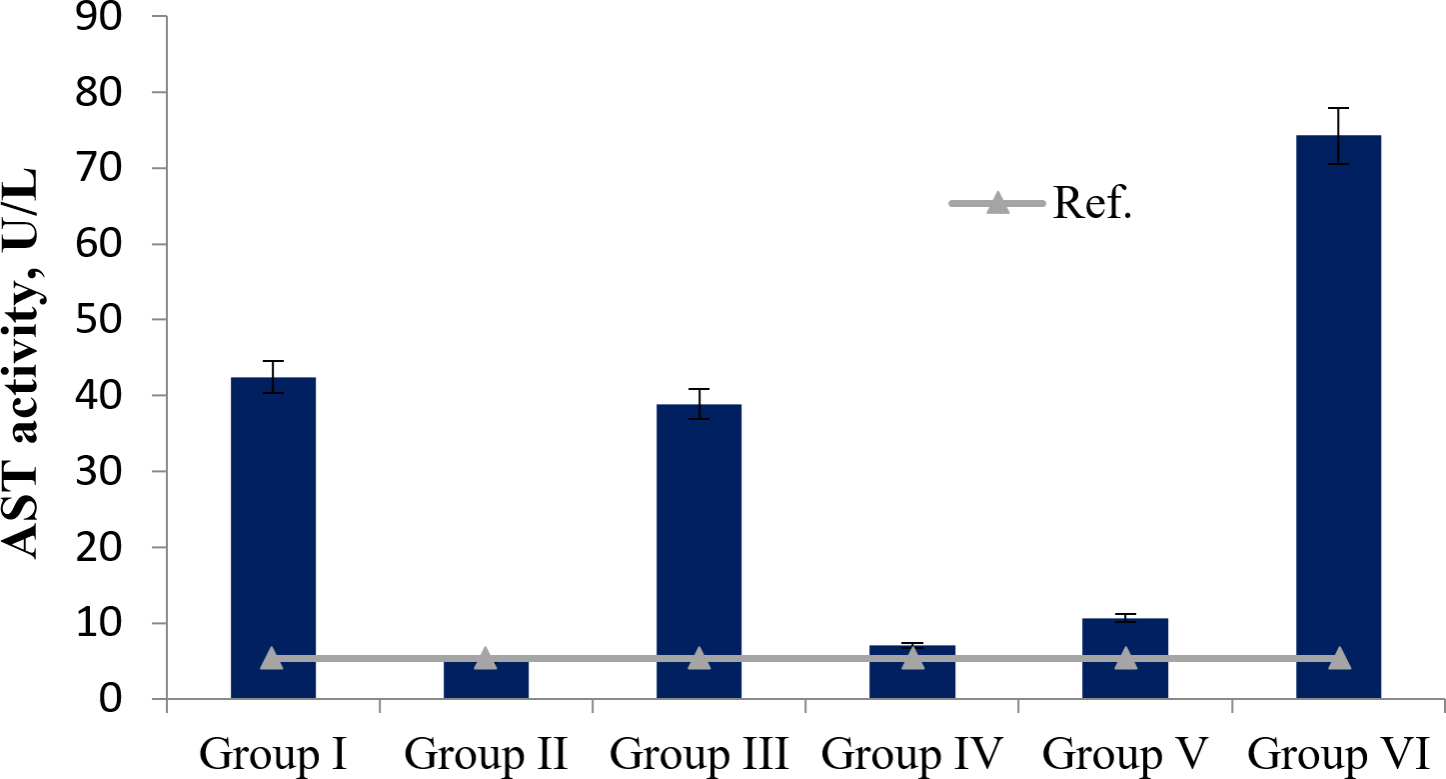
AST activity (U/L) in the plasma of all groups of experimental animals (*p* < 0.05).

The evaluation of hepatotoxicity based on alanine aminotransferase (ALT) activity values revealed no direct hepatotoxicity from the agents tested in groups I (3.54 ± 0.7 U/L), II (7.07 ± 0.7 U/L), V (3.54 ± 0.7 U/L), and VI (3.54 ± 0.7 U/L) compared to the control (for details on the compounds administered to each group, see Experimental section, “Animal experiment model”). However, co-administration of *T. polium* extract and Fe_3_O_4_ NPs (group III), as well as of rutin and Fe_3_O_4_ NPs (group IV), resulted in increased ALT activity relative to normal values (up to 10.6 ± 0.7 U/L) by 2.1 and 3.3 times, respectively (Figure 1). Also, increases in aspartate transaminase (AST) activity relative to control values (5.3 ± 0.3 U/L) were observed in groups I (8.01 times), III (7.34 times), V (2.00 times), and even in group VI (14.01 times), with values of 42.4 ± 0.3 U/L, 38.9 ± 0.3 U/L, 10.61 ± 0.3 U/L, and 74.26 ± 0.3 U/L, respectively (Figure 2).

**Figure 3:**
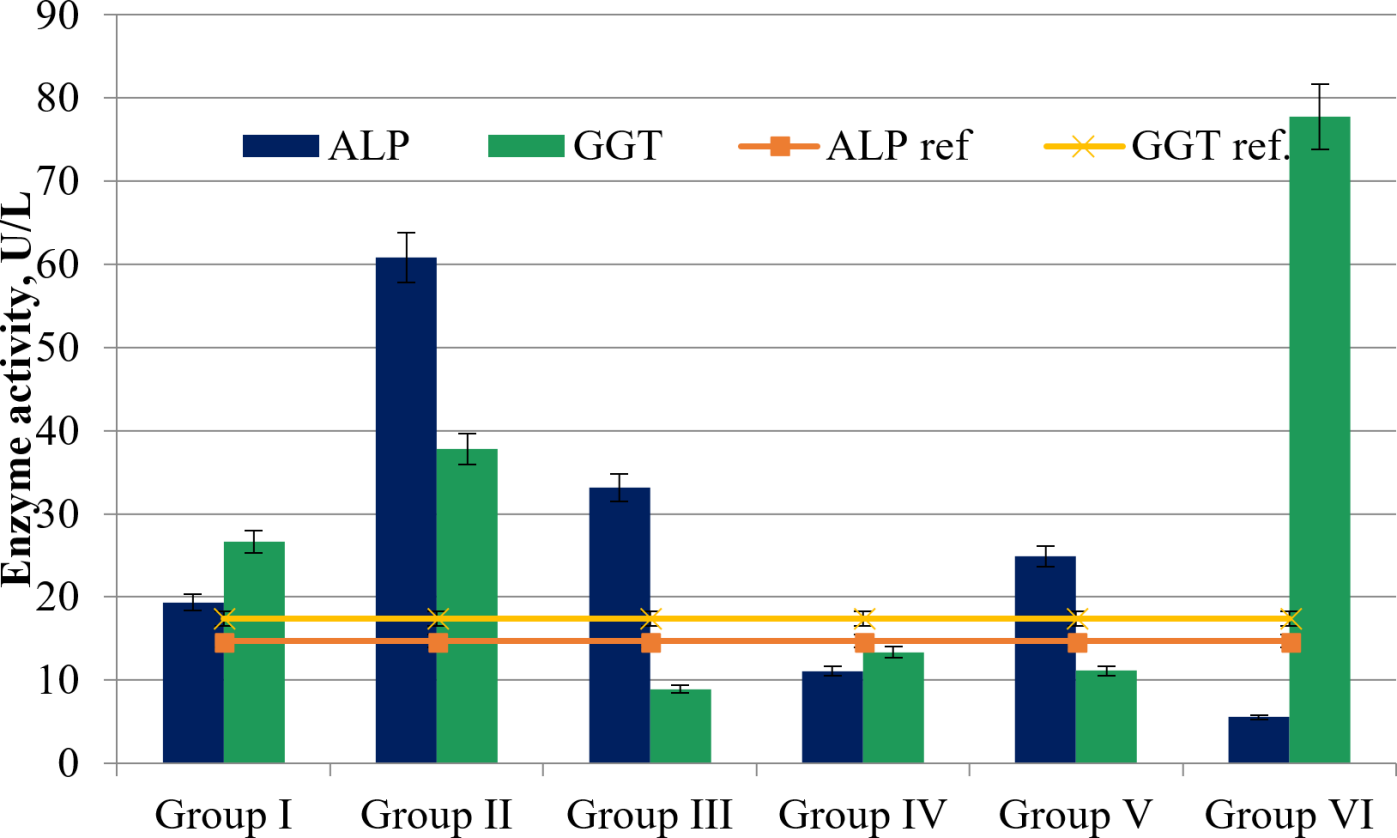
ALP and GGT activity (U/L) in the plasma of all groups of experimental animals (*p* < 0.05).

It can also be stated that in all groups, except for IV (11.06 ± 0.01 U/L) and VI (5.53 ± 0.03 U/L), there is an increase in alkaline phosphatase (ALP) activity (Figure 3, normal range 14.7 ± 0.17 U/L). The greatest increase in enzyme activity is observed in group II (4.14 times), while a slightly lesser increase is observed in group III (2.26 times). Against the backdrop of normal γ-glutamyl transferase (GGT) activity values (17.42 ± 0.63 U/L), groups III (8.89 ± 0.43 U/L) and V (11.11 ± 0.07 U/L) show normal levels. However, in group IV, where the agents involved were Fe_3_O_4_ NPs with rutin, there is no increase observed in either ALP (11.06 ± 0.01 U/L) or GGT (13.33 ± 0.14 U/L).

### Assessment of the effect on lipid metabolism

In the plasma of male Wistar rats in the control group, the normal level of total cholesterol reaches 2.87 ± 0.16 mM/L, HDL cholesterol is 0.8 ± 0.09 mM/L and higher, and LDL cholesterol is up to 2.07 ± 0.22 mM/L. According to these indicators, only in group I is the total cholesterol content close to normal values (Table 1). In the other groups, hypolipidemia is observed, with the maximum decrease in group IV. However, in some groups, there is an increase in HDL cholesterol (groups II, III, and VI), while in others, there is a decrease in HDL cholesterol (groups I, IV, and V), with almost identical minimal values in groups IV and V.

**Table 1:** Biochemical parameters of liver function in the plasma of experimental animals (*p* < 0.05).

	Control	Group I	Group II	Group III	Group IV	Group V	Group VI

total cholesterol, mM/L	2.87 ± 0.16	2.1 ± 0.12	1.54 ± 0.06	1.26 ± 0.05	0.62 ± 0.04	1.01 ± 0.02	1.11 ± 0.03
HDL, mM/L	0.8 ± 0.09	0.58 ± 0.03	1.19 ± 0.04	1.16 ± 0.01	0.39 ± 0.03	0.34 ± 0.04	1.09 ± 0.03
LDL, mM/L	2.07 ± 0.22	1.52 ± 0.07	0.35 ± 0.01	0.100 ± 0.001	0.23 ± 0.02	0.67 ± 0.04	0.020 ± 0.001

### Assessment of the impact on liver synthetic function

In the control group of animals, the level of albumin is 29.33 ± 1.3 g/L, and the level of total protein is 45.23 ± 1.43 g/L. In the control series of experiments, hyperalbuminemia is observed in groups III, IV, V, and VI according to this indicator (Table 2). Against this background, almost all groups, except for the groups II and V, also show an increase in total protein content. Increases compared to almost normal albumin levels are characteristic in pathological conditions when the total protein fraction increases due to acute phase proteins. Increases in total protein content may also be due to the release of ALT, AST, ALP, and GGT enzymes into the bloodstream.

**Table 2:** Biochemical parameters of liver function in the blood plasma of experimental animals in the control series (*p* < 0.05).

	Control	Group I	Group II	Group III	Group IV	Group V	Group VI

total protein, g/L	45.23 ± 1.43	55.59 ± 0.17	44.09 ± 0.09	52.51 ± 0.13	47.67 ± 0.08	42.11 ± 0.18	54.12 ± 0.31
albumin, g/L	29.33 ± 1.3	28.39 ± 0.23	27.8 ± 0.35	34.14 ± 0.09	35.64 ± 0.14	31.74 ± 0.09	46.15 ± 0.26

### Assessment of glutathione peroxidase activity

**Figure 4:**
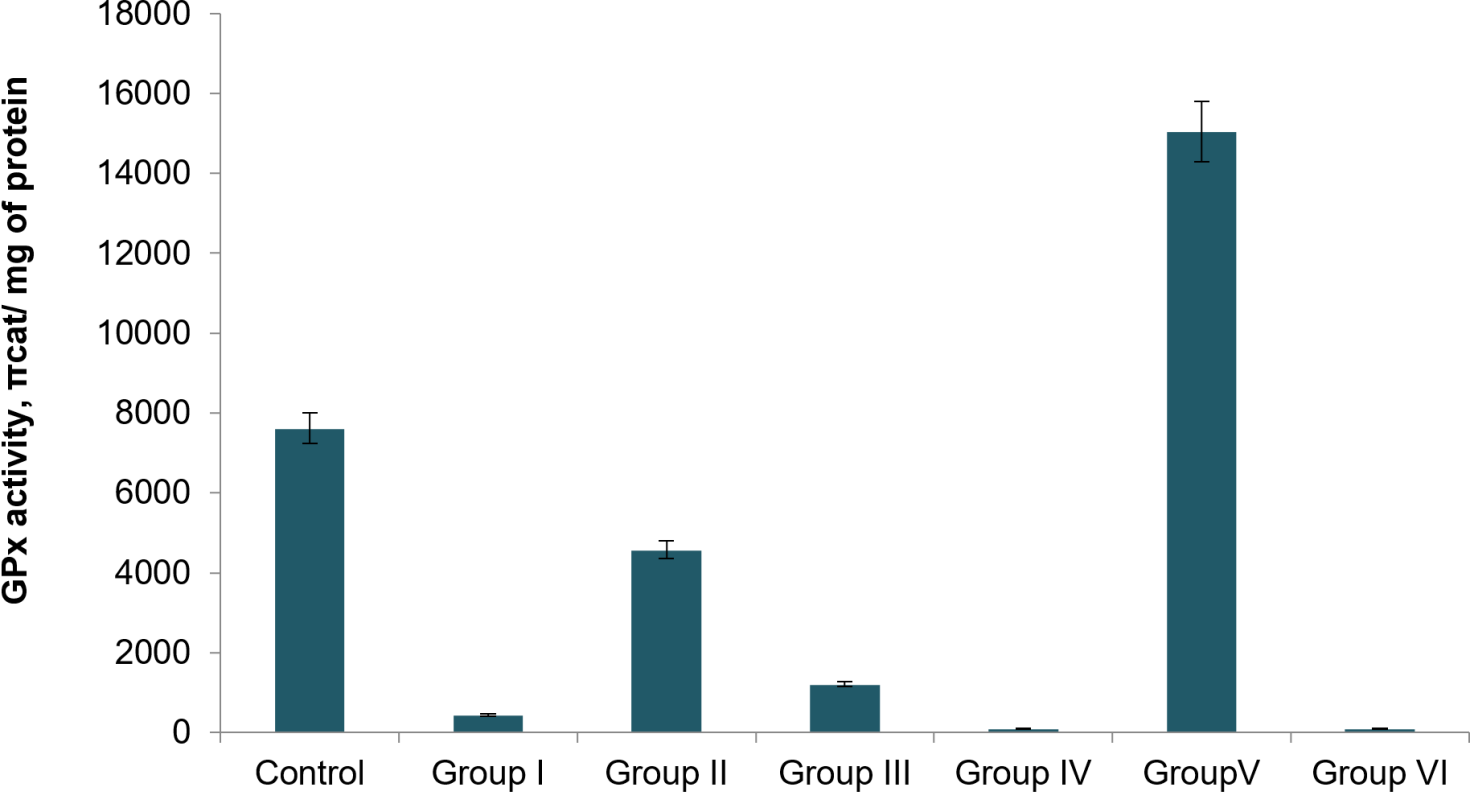
Activity of GPx in liver homogenates of experimental animals (*p* < 0.05).

The highest activity of glutathione peroxidase (GPx) was detected in the liver homogenates of group V rats, where the enzyme activity was 15052 ± 364 pkat/mg protein. This activity was 1.98 times higher compared to the GPx activity in healthy animal controls (up to 7624 ± 281 pkat/mg protein, Figure 4). In all other groups, GPx activity was lower than in the control values. For instance, in group II, this value was 4581 ± 172 pkat/mg protein, which was 1.66 times lower than that of the control. When the components of these groups were jointly applied, that is, rutin with Fe_3_O_4_ NPs (group IV), GPx activity in the liver homogenates was almost absent. A similar pattern was observed in group I, where basal GPx activity was detected, not exceeding 896 ± 93 pkat/mg protein; in group VI, GPx activity in the homogenates was virtually absent.

### Histological analysis of morphological changes in liver tissue

**Figure 5:**
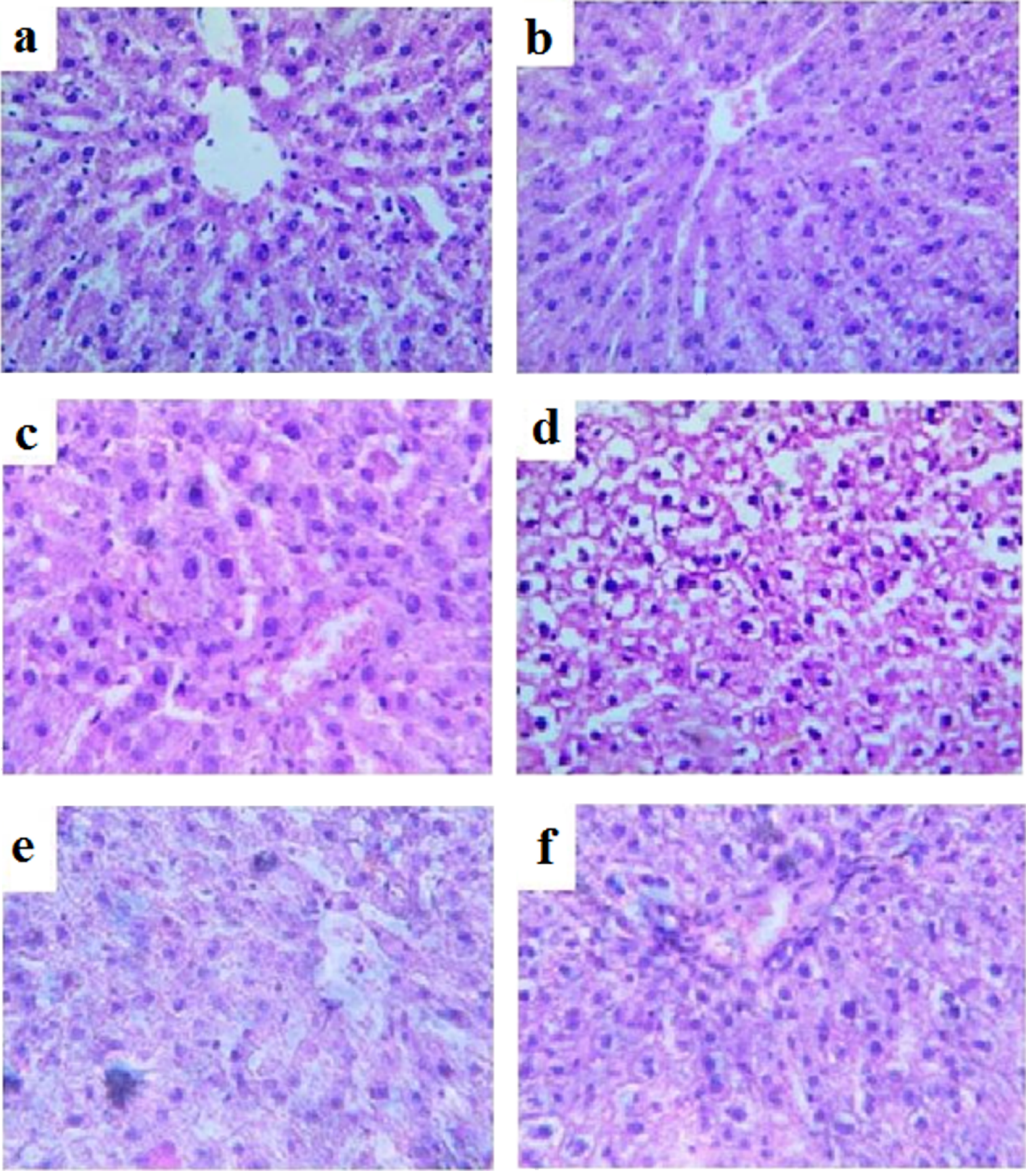
Microphotographs (ocular 20×, objective 100×) of liver tissue specimens from animals. (a) *T. polium* extract, (b) rutin, (c) *T. polium* extract with Fe_3_O_4_ NPs, (d) rutin with Fe_3_O_4_ NPs, (e) Fe_3_O_4_ NPs, and (f) “Karsil”.

In the histological examination of the toxic effects of the agents in all groups, a characteristic architecture typical of the liver was observed, with preserved lobular structure and radial arrangement of hepatocytes, as well as normal blood vessel filling (Figure 5).

However, in groups II and V, mildly pronounced dystrophic changes are observed in individual hepatocytes, that is, cytoplasmic opacity and the presence of optically distinguishable vacuoles; whereas in group IV, these dystrophic changes are observed in the majority of hepatocytes.

## Discussion

Combining plant extracts with biocompatible nanoparticles allows for the delivery of biologically active components to the target site with a reduced likelihood of their biotransformation, thus significantly lowering the therapeutic dose of these agents [52–54]. Moreover, nanoparticles themselves contribute to the overall observed effect because of their specific spectrum of biological activities. Therefore, the development of such complexes and the identification of their spectrum of biological properties are relevant issues in biomedicine with great potential for practical applications.

Spectral analysis of the composite of Fe_3_O_4_ NPs with 70% ethanol extract of *T. polium* in our previous studies revealed the formation of a complex with leveling of peaks characteristic of the extract and a hypochromic shift in the Fe_3_O_4_ NPs absorption spectrum [50].

The results of the impact of Fe_3_O_4_ NPs on liver tissue revealed practically no direct hepatotoxic properties. Increases in ALT and AST activities in some groups (Figure 1 and Figure 2) may be associated with effects of these agents also on the mitochondria of other cell types. Toxicity in cells leads to weakened mitochondrial activity, membrane leakage, and morphological changes. Toxic NPs can adversely affect cell viability, proliferation rate, and metabolic activity; also, they can reduce the therapeutic efficiency of the treatment [55]. The toxicity of NPs on biological entities fundamentally depends on the characteristics of the NPs, their dose, and the application [18]. Thus, based on ALT/AST activity values, it can be concluded that Fe_3_O_4_ NPs do not exhibit pronounced toxic effects on the livers of healthy Wistar rats; however, when co-administered with 70% ethanol extract of *T. polium* and rutin, changes occur in the activities of the formed complexes.

In all groups, except IV and VI, there is an increase in ALP activity (Figure 3). This enzyme exists in the body in the form of five different isoforms with different localizations (liver, bile ducts, kidneys, bones, and placenta). Normally, plasma activity reflects only the hepatic isoform (14.7 ± 0.17 U/L). However, against the backdrop of normal ALT values, an increase in ALP activity typically indicates a stagnation process in the bile ducts or a pathological process in the kidneys or bone tissue [56]. Comparing these data with the results of GGT activity determination (normal 17.42 ± 0.63 U/L) allows for determining the genesis of increased ALP activity (Figure 3). Simultaneous elevation of both enzyme activities indicates a localization of the pathological process in the bile ducts.

Against the backdrop of normal GGT activity in groups III and V, the increase in ALP activity may indirectly indicate the presence of a pathological process in other organs. Considering the fact that groups III and V received a combined administration with Fe_3_O_4_ NPs and the fact that many authors report the ability of these NPs to penetrate bone tissue, it can be assumed that the increase in plasma ALP activity is due to the bone isoform of this enzyme [56–57]. However, in group IV, where only Fe_3_O_4_ NPs with rutin were administered, no increase in either ALP or GGT was observed. Furthermore, according to literature data, the action of Fe_3_O_4_ NPs is dose-dependent, and Fe_3_O_4_ NPs (20–30 nm) administered to rat livers at doses up to 75 µg/g did not result in statistically significant changes in ALT, AST, and ALP activities [58].

Among the most important processes occurring in the liver are lipid metabolism and lipoprotein synthesis, which characterize its functional state. Based on the results showing a hypolipoproteinemic effect (Table 1), we hypothesize that one of the possible intracellular molecular targets of such action of Fe_3_O_4_ NPs and its composites are proteins of the SIRT family, regulating the activity of PPARγ, PGC-1α, NF-kB, FOXO, p53, and others, as well as SREBP1c, whose increased activity in liver tissue leads to hypoactivation and suppression of cholesterol and triglyceride synthesis, as well as hyperexpression of GPx [59].

The increase in protein and albumin content in the plasma of experimental animals may be associated with the ability of xenobiotics to enhance the expression of transport proteins during prolonged exposure, correlating with studies by Belinskaia and colleagues [60].

The combined effect of Fe_3_O_4_ NPs with 70% ethanol extract of *T. polium* and its component rutin leads to suppression of GPx activity, which may be associated either with the loss of this activity in the complex or with interaction with endogenous GPx and suppression of its antioxidant properties (Figure 4). Fe_3_O_4_ NPs themselves are capable of increasing GPx activity, likely because of their peroxidase-like enzymatic properties (group V) [19]. This scenario correlates with literature data on the peroxidase-like enzymatic properties exhibited by Fe_3_O_4_ NPs, which is reflected in the preservation of liver tissue morphology, but accompanied by changes in liver function biochemical markers (Figures 1–3, Table 1 and Table 2).

Considering the duration of exposure and the presence of noticeable dystrophic changes only in the group with maximum GPx suppression, it can be assumed that Fe_3_O_4_ NPs in combination with certain plant secondary metabolites may lead to excessive formation of free radicals, which cause dystrophic changes.

## Conclusion

The diversity of the obtained results is explained by the ability of Fe_3_O_4_ NPs to increase the bioavailability and intracellular concentration of the associated compounds, thereby reducing their therapeutic dose and altering the profile of dose-dependent effects. Additionally, both Fe_3_O_4_ NPs and their composites are presumed to interact with various spectra of intracellular structural regulators of biochemical processes.

Rutin and Fe_3_O_4_ NPs, individually, do not exhibit direct hepatotoxicity upon intraperitoneal administration in rats, unlike their complexes. Regarding lipid profiles, all agents tested induce a hypolipidemic effect through a reduction in LDL cholesterol, despite preserving synthetic functions of the liver. Fe_3_O_4_ NPs enhance GPx activity, which is attributed to their peroxidase-like enzyme-mimetic properties, while concurrently preserving the architecture of liver tissue.

Taking the above into account, it can be concluded that the effects of all investigated samples on liver tissue exhibit a multifaceted character and diverse mechanisms of activity. These mechanisms depend on the size of Fe_3_O_4_ NPs, the hydrophilic shell diameter, solubility, aggregation, stability, and various other physicochemical and biological parameters. Given the importance and relevance of the issue, each of these factors requires close attention and further investigation. However, Fe_3_O_4_ NPs of spherical shape with a diameter ranging from 4 to 24 nm at a dose of 40 mg/kg may be recommended for further in vivo studies.

## Experimental

### Synthesis and characterization of iron oxide nanoparticles

The synthesis of iron oxide (Fe_3_O_4_) nanoparticles was carried out using a modified coprecipitation method with oleic acid as a stabilizer. 10 mL of 1 M FeSO_4_·7H_2_O and 10 mL of 2 M FeCl_3_·6H_2_O were added to 10 mL 4 M NaOH and 1 mL oleic acid. Then, the mixture was thoroughly mixed and heated at 80 °C for 1 h until the color of the mixture turned from brown to black. The resulting black precipitate of Fe_3_O_4_ NPs was washed three times with deionized water and then dispersed for 40 min in an ultrasonic disintegrator (Ultrasonic Homogenizer Sonic-150W, MRC, Israel) at 80% power with an on/off cycle of 5/4 s. During the synthesis of the nanoparticles, a black precipitate with paramagnetic properties formed. Nature and morphology of this precipitate were determined using electron diffraction and transmission electron microscopy (TEM, LEO-912 abomega, Carl Zeiss, Germany) [50–52].

### Preparation of plant extracts

*T. polium* were collected in August–September of 2016 in Kotayk marz (40°16′40″ N, 44°39′49″ E, height above sea level: 1445 m), Armenia (collection of the Department of Medical Biochemistry and Biotechnology). After a preliminary wash and sterilization in 1% calcium hypochlorite solution (Sigma-Aldrich, Germany), plants were dried to 10% moisture level and ground in a mechanical homogenizer to obtain a homogeneous powder.

The method for obtaining *T. polium* extracts is similar to the extraction method of *Ocimum araratum* described in [61]. For the preparation of extracts, the plant powder was mixed with the extragents, that is, 96%, 70% and 50% ethanol, diluted in phosphate buffer (pH 7.4) in a 1:30 (w/v) ratio and exposed to ultrasound at 75 W (ultrasonic homogenizer, Sonic-150W, MRC, Israel). After 24 h of incubation on an orbital shaker (60–70 rpm), the mixture was centrifuged for 15 min at 3000 rpm (Jouan GR412, France) [50].

### HPLC analysis of plant extracts

To determine the major polyphenols, HPLC was employed using a Waters Alliance 2695 chromatograph with a spectrophotometric diode array detector, as well as MassLynx data processing software. Separation was done on a C-18 column (Knauer, Germany, 250 × 4 mm, particle size 4.5 nm) at an elution rate of 1 mL/min with the following gradient elution system: HPLC-grade water with 0.1% orthophosphoric acid (solution A, Carlo Erba, France), acetonitrile (solution B, Carlo Erba, France); 0–5 min linear, solution A was adjusted from 10% to 40%, then to 50% over the next 3 min; for the last 12 min, the ratio of solutions was maintained isocratic. Ethanol solutions of quercetin (Sigma-Aldrich, Germany), rutin (Sigma-Aldrich, Germany), apigenin (Sigma-Aldrich, Germany), kaempferol (Sigma-Aldrich, Germany) λ = 365 nm, naringenin (Sigma-Aldrich, Germany) λ = 290 nm, genistein (Sigma-Aldrich, Germany) λ = 261 nm and RA (Alfa Aeser, Germany) λ = 331 nm were used as standards [50].

### Antiradical activity of extract

Antiradical activity (ARA) of the extract was measured by the colorimetric method using the stable radical 2,2-diphenyl-1-picrylhydrazyl (DPPH, Alfa Aeser, Germany) at 30 °C. Optical density (OD) detection was carried out at 517 nm wavelength (UV-VIS 18, MRC, Israel), and ARA was estimated according to the following equation:




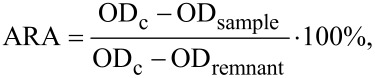




where OD_c_ is the optical density of the control, OD_sample_ is the optical density of the sample, and OD_remnant_ is the remnant optical density of DPPH after its complete scavenging.

The IC_50_ value was determined from dose-dependent ARA curves as the concentration of components in the sample necessary for quenching 50% of the DPPH radicals [62].

### Spectral analysis of sample complexes

The analysis of the spectra of the studied samples of 70% ethanol buffer extract of *T. polium*, rutin, and their complexes with Fe_3_O_4_ NPs was carried out at wavelengths from 200 to 800 nm using a UV–vis spectrophotometer UV-VIS 18 (MRC, Israel) [50].

### Animal experiment model

Experiments were conducted on mature male white Wistar rats weighing 190–210 g. The animals were kept on a regular diet and water regimen at a temperature of 25 ± 2 °C, 55% ± 5% humidity, and a 12 h day/night cycle. The animals were randomly divided into seven experimental groups of five to six individuals each, with intraperitoneal administration every three days for two months of 100 μL of the agents described in Table 3.

**Table 3:** Agents administered in the animal experiment model.

Group	Administered agents

control	70% ethanol buffer solution
group I	70% ethanol buffer extract of *T. polium* standardized by rutin content (1.87 μg/kg)
group II	70% ethanol buffer solution of rutin (1.87 μg/kg)
group III	complex of Fe_3_O_4_ NPs (40 mg/kg) with 70% ethanol buffer extract of *T. polium*
group IV	complex of Fe_3_O_4_ NPs (40 mg/kg) with rutin (1.87 μg/kg)
group V	70% ethanol buffer suspension of Fe_3_O_4_ NPs (40 mg/kg)
group VI	drug “Karsil” (7 mg/kg)

A non-toxic dose of Fe_3_O_4_ NPs of 15 ± 4 nm size was experimentally determined, considering the maximum permissible dose of 40 mg/kg in male laboratory mice [62]. All solutions of the agents were administered after being passed through antibacterial membrane filters (pore diameter 0.45 μm).

All interventions were performed in accordance with the principles of laboratory animal care of the Ethics Committee of Yerevan State Medical University (Yerevan, Armenia) and in accordance with the decision of 22 September 2010 of the Council of European Communities [2010/63/EU] and with the “ARRIVE” guidelines (Animals in Research: Reporting *In Vivo* Experiments). Euthanasia procedures were consistent with the recommendations of the American Veterinary Medical Association (AVMA) Guidelines on Euthanasia, using intraperitoneal injection of 70% ethanol [61].

For further biochemical analyses, blood was collected from the portal vein into vacutainers with the anticoagulant Na_2_EDTA. For histological studies, part of each liver tissue sample was fixed in 10% formalin, and another part was placed in 10 mM K/P buffer (pH 7.2) at 4 °C to study peroxidase enzyme activity.

### Determination of biochemical markers of liver function

Biochemical analysis to determine the activities of alanine aminotransferase (ALT; EC 2.6.1.2), aspartate aminotransferase (AST; EC 2.6.1.1), γ-glutamyl transferase (GGT; EC 2.3.2.2), and alkaline phosphatase (ALP; EC 3.1.3.1) in blood plasma, as well as the content of total protein (TP), albumin, total cholesterol (TC), HDL, and LDL, was carried out using standard BioSystems reagent kits (Barcelona, Spain) on a UV–vis spectrophotometer UV-VIS 18 (MRC, Israel) according to the attached BioSystems protocols.

### Determination of glutathione peroxidase activity in liver tissue

To determine the activity of liver glutathione peroxidase (GPx) (EC 1.11.1.9), tissue samples weighing 1–2 g were homogenized in 10 mM potassium phosphate buffer (pH 7.2) on ice using a Potter–Elvehjem manual homogenizer. The tissue homogenate was then centrifuged for 15 min at 8000*g* and 4 °C. The resulting supernatant was used to determine liver GPx activity [63]. GPx activity was determined by detecting the accumulation of the pyrogallol oxidation product, purpurogallin, at 420 nm on a UV–vis spectrophotometer UV-VIS 18 (MRC, Israel). Optical density was measured every 20 s for 3 min. The enzymatic mixture contained 0.8 mL of 10 mM K/P buffer (pH 7.2), 1.1 mL H_2_O, 0.5 mL of 0.15% H_2_O_2_, and 0.5 mL of 2 mM pyrogallol. The enzymatic reaction was initiated by adding 0.12 mL of the liver tissue homogenate supernatant. GPx activity was defined as the formation of 1 mg of purpurogallin from pyrogallol in 20 s.

Liver GPx activity was calculated using the formula:




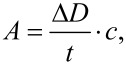




where *A* is the enzyme activity in pkat, Δ*D* is the change in optical density, *t* is the measurement time in seconds, and *c* is the protein concentration. Protein concentration in the liver tissue homogenate supernatant was determined using the method described in [61].

### Histological analysis of liver tissue

Liver tissue samples, previously fixed in 10% formalin, dehydrated, and embedded in paraffin, were sectioned into 5 μm slices using a microtome. The liver tissue sections were stained with hematoxylin and eosin according to standard techniques [64]. The histological preparations were analyzed using light microscopy on an AmScope microscope (USA), and microphotographs of the liver tissue samples were obtained using an AmScope MU500 5MP digital camera [61].

### Statistical analysis

Statistical analysis was based on the comprehensive application of standard statistical methods, including the calculation of mean values, standard deviations, and standard errors of the mean. Biological replicates ranged from four to six, with two to three series of experiments conducted for each. Tables, graphs, and diagrams present arithmetic means and their standard errors. The data are presented as mean ± SD. Statistical analysis of the results was performed using one-way analysis of variance (ANOVA). Student test (*t*-test) was used to compare data between groups. Differences and correlations were considered significant at *p* < 0.05.

## Data Availability

All data that supports the findings of this study is available in the published article and/or the supporting information to this article.
